# Evidence for gene flow and trait reversal during radiation of Mexican Goodeid fish

**DOI:** 10.1038/s41437-024-00694-1

**Published:** 2024-06-10

**Authors:** Leeban H. Yusuf, Yolitzi Saldívar Lemus, Peter Thorpe, Constantino Macías Garcia, Michael G. Ritchie

**Affiliations:** 1 Centre for Biological Diversity, School of Biology, University of St Andrews, St Andrews, UK; 2 Department of Biology, Texas State University, San Marcos, TX USA; 3 School of Life Sciences, University of Dundee, Dundee, UK; 4 Instituto de Ecologia, Universidad Nacional Autónoma de México, Ciudad Universitaria, Circuito exterior s/n anexo al Jardín Botánico C. P. 04510, Mexico City CdMx, Mexico

**Keywords:** Phylogenetics, Speciation

## Abstract

Understanding the phylogeographic history of a group and identifying the factors contributing to speciation is an important challenge in evolutionary biology. The *Goodeinae* are a group of live-bearing fishes endemic to Mexico. Here, we develop genomic resources for species within the *Goodeinae* and use phylogenomic approaches to characterise their evolutionary history. We sequenced, assembled and annotated the genomes of four *Goodeinae* species, including *Ataeniobius toweri*, the only matrotrophic live-bearing fish without a trophotaenia in the group. We estimated timings of species divergence and examined the extent and timing of introgression between the species to assess if this may have occurred during an early radiation, or in more recent episodes of secondary contact. We used branch-site models to detect genome-wide positive selection across *Goodeinae*, and we specifically asked whether this differs in *A. toweri*, where loss of placental viviparity has recently occurred. We found evidence of gene flow between geographically isolated species, suggesting vicariant speciation was supplemented by limited post-speciation gene flow, and gene flow may explain previous uncertainties about Goodeid phylogeny. Genes under positive selection in the group are likely to be associated with the switch to live-bearing. Overall, our studies suggest that both volcanism-driven vicariance and changes in reproductive mode influenced radiation in the *Goodeinae*.

## Introduction

Genomic analyses are providing unprecedented insights into evolutionary history, demography, levels of introgression, and the identification of key genes under selection during evolutionary divergence. Vicariance models of speciation (Coyne and Orr 2004; Mayr 1963) still predominate our understanding of speciation history, but the precise timing (especially recent Pleistocene versus older splits) and levels of introgression during speciation have been revised following genome scale analyses. Many species groups show biogeographic patterns consistent with recent vicariant divergence during the Pleistocene (Schmitt 2007; Kadereit and Abbott 2021), but initial divergence may have deeper roots and have begun earlier (Klicka and Zink 1997; Ebdon et al. 2021). The “repeated allopatry and secondary contact” models of speciation (Harrison and Larson 2014; Suvorov et al. 2022) suggest that opportunities for introgression may be common and gene flow is now commonly inferred from genomic comparisons. Indeed, gene flow can facilitate speciation in multiple ways (Marques et al. 2019), for example, hybridisation can provide genetic material contributing to adaptive radiation, either by increasing genetic variation or promoting exchange of adaptive genotypes. Some of the most detailed studies of this concern freshwater lacustrine radiations, especially of the African cichlids (Meier et al. 2017; Malinsky et al. 2018; McGee et al. 2020; Svardal et al. 2020). Determining the timing and biological significance of gene flow during speciation in species groups with complex evolutionary histories is therefore an important challenge.

Central Mexico is a biodiversity hotspot, but relatively few detailed phylogeographic studies of endemic groups have been completed. Pleistocene climatic fluctuations are often invoked to explain biogeographic patterns, but these have been described as “complex” in comparison with temperate regions (Colin and Eguiarte 2016). Central Mexico contains high altitude upland and has been subject to extensive dynamic volcanism during the Miocene and Pleistocene (Ferrari et al. 2012). The Trans-Mexican volcanic belt is implicated in a Mexican Transition Zone where Nearctic and Neotropical biotas meet (Morrone 2010). The highlands may be characterised by sky islands with frequent fragmentation. Mastretta-Yanes et al. (2015) argued that such changes may have led to opportunities for hybridisation during speciation of Mexican highland species. Freshwater fish, as well as other organisms (Musher et al. 2022) associated with river networks, may have an added complexity to their distributional changes as relatively recent volcanism has influenced river patterns (Dias et al. 2013; Craw et al. 2016), including river piracy. In *Poeciliopsis* and *Poecilia*, phylogeographic studies implicate a Plio-Pleistocene vicariant event in driving speciation, stimulated by the Trans-Mexican Volcanic Belt (Mateos 2005). Other biogeographical studies show concordant patterns of river vicariance driven by volcanic activity in the Trans-Mexican Volcanic Belt in less well-studied freshwater fish taxa (Domínguez-Domínguez et al. 2006; Pérez-Rodríguez et al. 2015).

The Goodeidae are a group of freshwater fish that vary in reproductive mode. They consist of the oviparous *Empetrichthyinae*, from Nevada and California and the viviparous *Goodeinae*, comprising 36 species across 16 genera confined to Mexico. Populations of most species within *Goodeinae* have declined precipitously over the last 20 years, with some already declared extinct in the wild or critically endangered (Lyons et al. 1998, 2019; Domínguez-Domínguez et al. 2008; Dominguez-Dominguez et al. 2010), and population genetic analyses of wild and captive species show evidence of population decline and inbreeding in natural populations (Bailey et al. 2007). Previous studies, mainly based on mtDNA, have suggested that the Goodeinae radiated in the Miocene ~16 MYA predominantly due to allopatric speciation (Webb et al. 2004; Doadrio and Domínguez 2004; Foster and Piller 2018). Their helminth parasites show similar biogeographic patterns (Quiroz-Martínez and Salgado-Maldonado 2013). The phylogeny is not unambiguously resolved and is usually divided into a number of tribes, from three to five (summarised in Caballero-Viñas et al. (2023). Recent studies adding nuclear markers have failed to resolve some relationships with conflict between markers (Parker et al. 2019).

The Goodeinae may represent an adaptive radiation, but multiple features may have influenced this, including the evolution of live-bearing, sexual selection, or ecological radiation. All species within the Mexican *Goodeinae* are viviparous and matrotrophic, with rapid offspring development dependent on maternal provisioning (Wourms et al. 1988; Vega-López et al. 2007) via a placental analogue called the trophotaenia (Wourms et al. 1988; Hollenberg and Wourms, 1995). Within the *Cyprinodontiformes* (including the *Goodeidae* and *Poecilidae*) speciation rate is higher in live-bearing fish (Helmstetter et al. 2016), so genes associated with this reproductive mode might be expected to be key elements in the radiation of the *Goodeinae*. Interestingly, one species of Goodeid, *Ataeniobius toweri*, is thought to have lost the trophotaenia, though little is known about any physiological or genetic changes involved. *Ataeniobius* is also subject to phylogenetic uncertainty as some studies include this genus and *Goodea* in the tribe Goodiini, whereas others find these genera to be distinctly placed in the phylogeny (Foster and Piller 2018). Body form has changed extensively, with increased sexual dimorphism in many species indicative of sexual selection, which may have increased speciation rate (Ritchie et al. 2005). But functional morphological ‘modules’ have also diverged rapidly compared to sister groups, possibly indicating ecological specialisation (Foster and Piller 2018).

We have obtained samples of four new *Goodeinae* genomes, including *A. toweri*, and analysed whole-genome sequence data for a total of eight species in the group. These include representatives from eight of the 16 genera within *Goodeinae*, capturing all the proposed tribes and major clades based on previous phylogenies (Webb et al. 2004) and the degree of matrotrophy across the group. Additionally, we have included whole-genome data for *C. baileyi*, an oviparous outgroup species in the other sub-family, *Empetrichthyinae*, to facilitate phylogenetic reconstruction and divergence dating of *Goodeinae*. We analyse divergence in these species and in particular, we ask:

- If the inferred split times are consistent with previous estimates, and if there is evidence of introgression.
- Which genes show evidence of positive selection in the group; are these associated with live-bearing?
- Which genes are associated with evolutionary reversal of trophotaenia development in *A. toweri* and is there any evidence of loss of function in key genes?

## Methods

### Sampling and whole-genome sequencing

Muscle tissue samples were obtained from one adult male per species *of Ameca splendens, Ilyodon furcidens, Characodon lateralis and A. toweri*. *A. splendens and I. furcidens* were descendants of fish captured in Jalisco state (Mexico). *C. lateralis* and *A. toweri* fish were collected in Durango and San Luis Potosí states, Mexico, respectively. Fish were captured under SEMARNAT permits SGPA/DGVS/00824/20, SGPA/DGVS/04507, SGPA/DGVS/09253, SGPA/DGVS/01290/13 and CONAPESCA permit DGOPA/12548/151104/4122. Fish of *C. lateralis* and *A. toweri* were captured using minnow traps. Captured fish were put into a bucket that contained local water, Stress Coat ® for reducing stress and potassium permanganate as antiseptic. They were subsequently transported to the Aquarium of the Institute of Ecology, UNAM (Mexico) and treated against any potential infection. Once those treatments were over, one male per species was ethically euthanized, and their tissues were treated with RNAlater following the manufacturer’s protocol (Thermo Fisher Scientific™) for long term storage. Subsequently, DNA was extracted from a piece of muscle of each male using Qiagen’s DNeasy Blood & Tissue Kit and following the kit’s instructions. DNA purity was measured with NanoDrop (Thermo Fisher Scientific™) and DNA concentration quantified with Qubit (Thermo Fisher Scientific™).

Extracted DNA from *I. furcidens, A. splendens, A. toweri and C. lateralis* was sent to Novogene (Beijing, China), for library preparation and sequencing. Sequencing libraries were generated using the NEBNext® DNA Library Prep Kit (New England Biolabs, USA). The genomic DNA was sheared to a size of 350 bp, then the fragments were end-polished, A-tailed, and ligated with the NEBNext adaptor (New England Biolabs™, USA) for Illumina sequencing. Libraries were analysed for size distribution with 2100 Bioanalyzer (Agilent™) and quantified using real-time PCR. Paired-end sequencing was performed on an Illumina NovaSeq 6000 system (Illumina™ Inc.) using the v1.0 reagents for sequencing.

### Read mapping and variant filtering

Raw genomic reads for each species were trimmed using fastp v.0.20.1 (Chen et al. 2018) with default parameters. Cleaned reads were then mapped to a *G. multiradiatus* reference genome (Du et al. 2022) using bwa (v.0.7.17). We used samtools (v. 1.11) (Li et al. 2009) to index, sort, mark and remove duplicate reads from each sample and Picard (Broad Institute 2019) to add read groups to each sample. Subsequently, variant calling was performed on mapped reads using Freebayes (v.1.3.2) and variants were filtered using GATK hard-filtering best practices guidelines (Van der Auwera et al. 2013). The hard filtered VCF file used for all subsequent analyses contained 1,884,779 SNPs.

### Multi-species gene alignments

We used the reference genome from *G. multiradiatus* (Du et al. 2022) and our hard-filtered variant calls to produce a consensus sequence alignment for each gene using the vcf2fasta software (https://github.com/santiagosnchez/vcf2fasta). To ensure gene alignments did not contain de-novo genes not found in our outgroup species *C. baileyi*, alongside the reference genome and hard-filtered VCF file, we provided a filtered gene annotation file containing only annotated genes found in both *G. multiradiatus* and in *C. baileyi*. To produce this consensus gene annotation, we lifted annotations from *C. baileyi* to the *G. multiradiatus* annotation using the programme liftoff (v.1.6.1) (Shumate and Salzberg 2021); only 20,138 out of 29,739 genes we considered. Nucleotide gene alignments were then aligned using MACSE v2.05 and the following parameters: “-prog refineAlignment -optim 2 -local_realign_init 0.001 -local_realign_dec 0.001”. Nucleotide alignments were then masked for any remaining internal stop codons and frameshift mutations using the parameters: “-codonForInternalStop NNN -codonForInternalFS --- -charForRemainingFS – ”. Finally, nucleotide alignments were translated into amino acid alignments and were subsequently used to produce a codon alignment using the programme PAL2NAL and the parameters: “-nogap -nomismatch”.

### Phylogenetic inference of divergence within *Goodeinae*

#### Maximum likelihood inference using IQTREE

To accurately infer the divergence times of the *Goodeinae*, we sought to further filter variants used in our analyses. Specifically, we removed variants with missing genotypes across any of our samples, non-biallelic variants, and variants with either no alternative allele or only alternative alleles using bcftools. Additionally, to ensure we sampled SNPs equally across the genome, we pruned SNPs by sampling 1 SNP for every 100 SNPs using the following parameters in bcftools: bcftools +prune -w 100 bp –n 1. The pruned and filtered VCF was then converted into a phylip alignment by concatenating all variants using vcf2phylip (Ortiz 2019) for all nine species. We first inferred phylogenetic relationships using an IQTREE2 (v.2.1.4) using ModelFinder (transversion model, AG = CT and equal base frequency (TMVe)(Nguyen et al. 2015; Kalyaanamoorthy et al. 2017) and 1000 bootstraps.

#### Species tree inference using SVDquartets

Since maximum-likelihood concatenation approaches may fail when incomplete lineage sorting is frequent, we sought to infer a species tree using SVDquartets (Chifman and Kubatko 2014, 2015) in Paup (v.4.0a) (Wilgenbusch and Swofford 2003) performing 100 bootstraps and using filtered, pruned variants.

#### Species tree inference using ASTRAL

Alongside SVDquartets, we also used ASTRAL, another coalescent-based approach, to infer the species tree (Mirarab et al. 2014), to examine consistency of results with different approaches. We utilised the 20,138 multi-species gene alignment as detailed above. For each alignment, gene trees were produced with IQTREE using a GTR + I + G model. Gene trees with non-zero branch lengths were retained leaving 19,908 inferred gene trees. ASTRAL (v.5.7.8) was then used (with default parameters) to infer a species tree using the 19,908 inferred gene trees, specifying *C. baileyi* as the outgroup.

#### Gene concordance analysis

Since classic measures of phylogenetic support tend to overestimate confidence in phylogenomic datasets (Kumar et al. 2012; Salichos and Rokas 2013; Yang and Zhu 2018), we estimated gene concordance factors using the species tree inferred with ASTRAL and 19,908 gene trees, via IQTREE2 with default parameters. Gene concordance factors quantify the percentage of gene trees that contain a given branch in the species tree (Minh et al. 2020).

#### Dating divergence in *Goodeinae*

To infer divergence times and effective population sizes for ancestral nodes, we used the A00 model in BPP (v.4.4.0) and putatively selectively-neutral non-coding loci (Flouri et al. 2018) (see Supplementary materials for full details).

#### Testing global and local patterns of introgression

To test for evidence of introgression between species, we calculated Patterson’s D-statistic using the *Dtrios* function in Dsuite v.4 (Green et al. 2010; Malinsky et al. 2020) with hard filtered variants (described above). The D-statistic examines site patterns in (minimally) four genome sequences of three in-group populations/species and an outgroup to test for a deviation from a strict bifurcating evolutionary history (Green et al. 2010; Durand et al. 2011). We tested for introgression using the D-statistic across 56 trios (total possible number of trios) arranged according to phylogenetic relationships in our inferred species tree. As well as calculating D-statistics for all 56 trios, we also calculated the admixture fraction (*f4-ratio*) (Reich et al. 2009). To control for false discovery, we applied a Benjamini–Hochberg correction to p-values for all trios and kept only those with corrected p-values lower than 0.05 (Benjamini and Hochberg 1995). To filter out trios with unreliable introgression signals, we removed those where *D* and the *f4-ratio* were lower than 1%. Additionally, we inferred the f-branch statistic via *Dsuite* to clarify whether introgression signals represent recent introgression between extant lineages or ancient introgression between ancestral lineages (see Supplementary material for more details).

To supplement tests for introgression, we also sought to understand local patterns of introgression and identify genes that may have been introgressed. We estimated f_d_ genome-wide in 150 bp windows using the *Dinvestigate* programme in *Dsuite* (Martin et al. 2015; Malinsky et al. 2020). Specifically, we surveyed introgression genome-wide only for trios that showed the most consistent signals of introgression across all genome-wide estimates of introgression. To determine genes which may have introgressed across trios, we subsetted our dataset to only include windows within the top 10% of windows based on f_d_ genome wide. We then used the *GenomicRanges* package (Lawrence et al. 2013) in R to determine whether any windows overlapped with annotated genes in our gff file. This subsetting approach allowed for the conservative identification of high-confidence introgressed genes maintained across multiple independent introgression events or an ancient event(s).

#### Detecting positive selection in rapid diversification of *Goodeinae*

We used two approaches to detect positive selection in internal branches leading to the rapid diversification of *Goodeinae*. First, for each internal branch and codon alignment, we asked whether, at a given branch, some sites were evolving under positive selection relative to background branches using aBSREL (Smith et al. 2015). For each codon alignment, we used the inferred species tree phylogeny. Since all internal branches were tested separately, p-values were corrected for multiple testing and filtered (*p* < 0.05) using the Benjamini–Holm procedure. Secondly, for all internal branches considered together, we tested whether specific codons in each codon alignment (gene) were under positive selection in internal branches, using the codon model MEME (Murrell et al. 2012) and the species tree phylogeny. Again, p-values were corrected for multiple testing using the Benjamini–Hochberg procedure (Benjamini and Hochberg, 1995). Extraction of results for aBSREL and MEME from JSON files was performed using Python package *phyphy* (Spielman 2018). Additionally, we performed gene ontology via the R package ‘clusterProfiler’ on genes showing evidence of positive selection on at least one internal branch (Yu et al. 2012).

To facilitate understanding how trophotaenia was lost in *A. toweri*, we performed a branch-site test via aBSREL on the terminal branch leading to *A. toweri*. We supplemented this test for positive selection in *A. toweri* in two ways. First, by overlapping positively-selected genes with genes upregulated in the trophotaenia of *G. multiradiatus*, taken from a recent genomic and transcriptomic analysis of *G. multiradiatus* (Du et al. 2022). Up-regulated genes were categorised as such if they showed log^2^ fold change larger than 2 in trophotaenia in comparison to all other tissue types (brain, testes, ovary, embryos) (Du et al. 2022). Second, we used the Ensembl Variant Effect Predictor (VEP) tool to infer, using genome annotation information, high-impact mutations in protein-coding genes that may drastically alter protein function (McLaren et al. 2016). Altogether, we searched for genes upregulated specifically in trophotaenia of *G. multiradiatus* that may also have high-impact mutations in *A. toweri* indicating potential pseudogenization.

## Results

### Rapid diversification of *Goodeinae* occurred in the middle Miocene

**Fig. 1:**
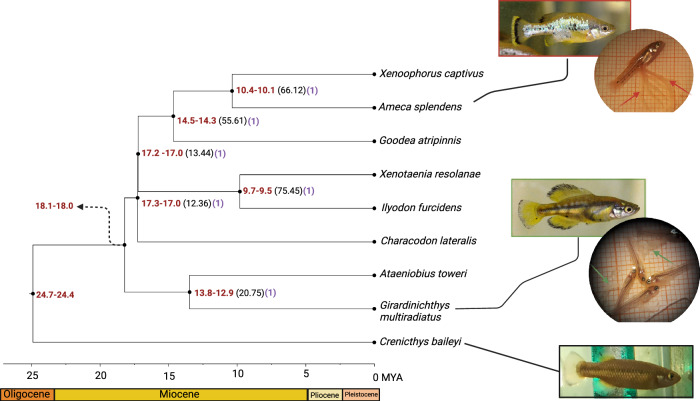
Relationships of the species of the family *Goodeinae* sampled in this study*.* Scaled divergence times are shown for each node (in red) and gene concordance factors are shown for relevant nodes (in black and in parentheses). Local posterior probability for each branch are shown in purple. Pictures show *C. baileyi*, *A. splendens* and *G. multiradiatus* alongside pictures of near-term embryos extracted from pregnant females of *A. splendens* and *G. multiradiatus*. Red and green arrows point towards exposed trophotaenia of *A. splendens* and *G. multiradiatus*, respectively (note the contrasting morphology).

Both coalescent approaches (ASTRAL and SVDquartets) to inferring the species relationships yielded a consistent topology (Fig. 1) with complete bootstrap support and posterior probabilities, and only partly recapitulate patterns inferred from some previous molecular phylogenies using mitochondrial and other sequences (Webb et al. 2004; Doadrio and Domínguez 2004; Foster and Piller 2018), summarised in Caballero-Viñas et al. (2023). We found that following the divergence of *Empetrichthyinae* (represented by *C. baileyi*), a group containing *A. toweri* and *G. multiradiatus* (tribe Girardichthyini) diverged next, supporting previous suggestions of an evolutionary reversal of trophotaenia complexity in *A. toweri*. In other analyses, the Ilyodontini (containing *I. furcidens* and *X. resolanae*) or Characodontini (containing *C. lateralis*) are the first tribes to diverge within Goodeinae (also supporting a scenario of trophotaenia reversal in *A. toweri*) (Webb et al. 2004; Doadrio and Domínguez 2004; Foster and Piller 2018). Our analysis suggests that *C. lateralis* diverged following the split of *A. toweri* and *G. multiradiatus* instead of diverging earlier. The remaining species fall into two groups: one containing *I. furcidens* and *X. resolanae (*Ilyodontini*)*, and another containing *G. atripinnis*, *A. splendens* and *X. captivus (*Girardichthini and Chapalichthyini) (Fig. 1). Our analysis does not support the existence of a ‘Goodiini’ tribe containing *Goodea* and *Ataeniobius*.

Although well supported, we found that almost all branches show extremely low gene concordance, except for branches leading to the split of *G. atripinnis*, *A. splendens* and *X. captivus*, and of *I. furcidens* and *X. resolanae* (Fig. 1). This gene tree discordance is confirmed by inference of normalised quartet scores in ASTRAL where only 60% of quartets across all gene trees are also found in the species tree. Additionally, around the crown radiation, we observe short branch lengths and broad variation in support for alternate topologies, indicating frequent incomplete lineage sorting, presumably as a result of rapid speciation of the *Goodeinae* (Fig. 1).

To understand when diversification of *Goodeinae* began, we estimated divergence times and effective population sizes using a filtered dataset of 2740 putatively neutral non-coding loci, the species tree estimated above using ASTRAL and a multi-species coalescent approach. We found that the crown age of the group and the divergence of *Empetrichthyinae* occurred between 24.7–24.4 MYA (Fig. 1 and Supplementary Table 1). We estimated that the diversification of *Goodeinae* began 18 MYA with the diversification of a clade containing *G. multiradiatus* and *A. toweri*. Following the split of *G. multiradiatus* and *A. toweri*, divergence of two clades containing (a) *G. atripinnis*, *A. splendens* and *X. captivus*, and (b) *I. furcidens* and *X. resolanae* occurred alongside divergence of *C. lateralis* around 17 MYA. We estimated that the split of *C. lateralis* from the other lineages was accompanied by considerable reduction (~100 fold) in effective population size, likely owing to vicariance driven by volcanism (Webb et al. 2004). This is supported by similar reduction (~5 fold) in effective population size following the split of *C. baileyi*. Divergence of *A. toweri*, where evolutionary reversal of trophotaenia is hypothesised, occurred around 13.9–13.1 MYA. Altogether, our divergence time and effective population size estimates suggest incomplete lineage sorting is likely explained by rapid vicariant speciation events that occurred early in the diversification of Goodeids.

### Limited evidence of introgression supports vicariant speciation of *Goodeids*

**Fig. 2:**
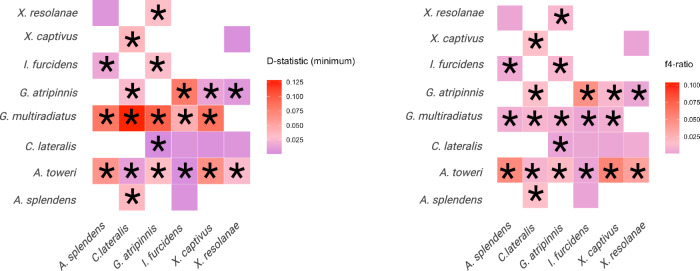
Evidence of gene flow (coloured panels) between species in *Goodeinae.* Plots showing D-statistics and f4-ratios in a pairwise manner. Significant allele sharing after multiple-testing correction is indicated with an asterisk. Empty coloured squares denote D-statistic and f4-ratio comparisons that were not significant after multiple testing.

Alongside incomplete lineage sorting, gene flow may also produce patterns of underlying gene tree conflict with the species tree. To assess the potential importance of gene flow in explaining patterns of genetic variation in Goodeids, we computed Patterson’s D statistic and f4-ratios for all possible species combinations. We found that 41 out of 56 tested trios (73%) have a significantly positive D-statistic value (D_min_) after correcting for multiple testing, with average genome-wide allele sharing of 4.7% (Fig. 2).

However, estimates of introgression are not independent since a single, ancestral gene flow event may result in erroneous interpretations of recent, widespread gene flow. We therefore re-calculated D_min_ by reorganising trios according to the relationships observed in the species tree inferred using ASTRAL. Specifically, we calculated the f-branch statistic ƒb(C) which utilises correlated f4-ratios to provide branch-specific estimates of introgression. We found support for gene flow events (ƒb(C) > 10%) between *A. toweri* and all other species apart from *G. multiradiatus* (Supplementary Fig. 1). Additionally, we found support for weaker introgression between *G. multiradiatus* and an ancestral branch leading to the divergence of *G. atripinnis, X. captivus* and *A. splendens*. Altogether, we suggest there is consistent evidence for an ancient gene flow event between the ancestor of *G. multiradiatus* and *A. toweri* and an ancestor of *G. atripinnis, X. captivus* and *A. splendens*.

**Fig. 3:**
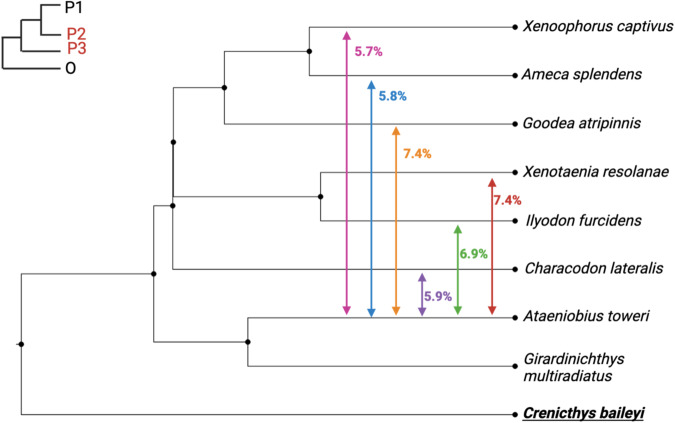
Mean genome-wide admixture proportions (f_d_) between *A. toweri (P2)* and other Goodeines (P3). *Crenicthys baileyi* used as outgroup species in all contrasts and *G. multiradiatus* was used as an in-group species (P1). Phylogeny created using Figtree.

To examine patterns of local introgression and identify of introgressed genes with high confidence, we computed f_d_ for the introgression event(s) with most support. We examined introgression between trios containing *G. multiradiatus* (P1), *A. toweri* (P2) and all other Goodeines (P3), with *C. baileyi* (P4) used as the outgroup (Supplementary Fig. 2). We found similar levels of mean admixture proportions across surveyed comparisons (Fig. 3), with highest levels of mean admixture (~7.4%) observed in *G. atripinnis*, which shares its range with *A. toweri*, and in *X. resolanae*, which is currently allopatric from *A. toweri*. In *C. lateralis* (which inhabits a northern, endorheic basin), *A. splendens* and *X. captivus*, lower levels of genome-wide admixture with *A. toweri* are observed (5.7–5.9%), perhaps owing to differential loss of introgressed variation.

Across all comparisons, we found 64 genes that show evidence of high f_d_ (top 10%) across all trios. Those genes include class II, major histocompatibility complex, transactivator (CIITA), which mediates expression of other MHC II genes and is under divergent sequence evolution in other viviparous lineages (Roth et al. 2020). We found a significant enrichment of genes related to sugar and fat metabolic processes, as well as activation and regulation of transcription factors (Supplementary Table 2). However, gene ontology analysis of introgressed genes maintained across all comparisons showed no significant enrichment of any term related to biological processes, after correcting for multiple testing. Since introgressed genetic variation may be lost by drift or removed by purifying selection, we also conducted gene ontology analysis on the contrast with most introgressed genetic variation (introgression between *A. toweri* and *X. resolanae*) to recover as much genetic variation that was initially exchanged as possible. We found significant enrichment of genes involved in cilium projection and organisation, cell projection and gastrulation (Supplementary Table 3).

### Positive selection across *Goodeinae*

**Fig. 4:**
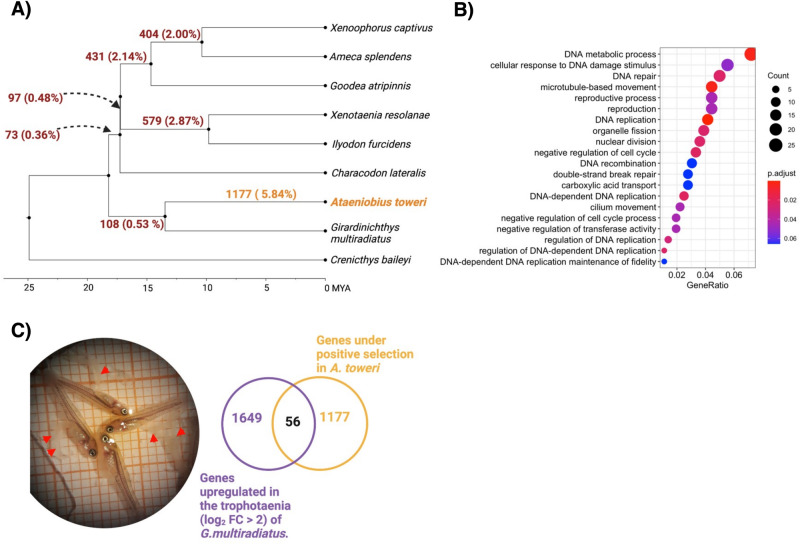
Testing for evidence of positive selection in branches across *Goodeinae.* **A** Phylogeny of *Goodeinae* showing the number of genes and the percentage of genes under positive selection at tested branches. **B** Gene ontology terms associated with genes under positive selection across *Goodeinae*. Colours denote adjusted *p*-value (for multiple testing) and size of data point denotes number of genes under positive selection. GeneRatio indicates the ratio of the number of genes under positive selection and the background gene set for each enrichment term. **C** Near term embryos extracted from a pregnant of *G. multiradiatus*. Red arrows point towards exposed trophotaenia. Venn diagram shows the number of genes upregulated in the trophotaenia of *G. multiradiatus*, the number of genes under positive selection in *A. toweri*, and their overlap.

To understand what genes may have been associated with diversification across *Goodeinae*, we performed branch-site tests in all internal branches in the species tree. After multiple testing correction, we found 1482 (~7.3% of all 20,138 genes tested) genes that were under positive selection in at least one internal branch. Of these 1482 genes, 1293 (~6.4% of all genes tested) are only under positive selection in a single branch, with 168 (~0.8%) genes showing evidence of positive selection in two branches and 21 (~0.1%) genes evolving under positive selection in three branches. Genes under selection across three internal branches include canonical immune response genes such as interleukin-2 receptor subunit gamma (*IL-2RG*), cluster of differentiation 4 (*CD4*) and Lymphocyte activation gene 3 (*LAG3*). Gene ontology across all positively selected genes (1482) shows a significant enrichment (FDR < 0.05) of genes involved in DNA damage and repair, reproduction and cilium movement, among others (Fig. 4; Supplementary Table 4).

Additionally, we examined the number and identity of genes under positive selection in each internal branch. In the branch leading to the split of *G. multiradiatus* and *A. toweri*, we found 108 genes under positive selection. In subsequent internal branches leading to the split of *C. lateralis* and the ancestor of *I. furcidens*, *X. resolanae*, *G. atripinnis*, *A. splendens* and *X. captivus*, we found even fewer genes under positive selection (73 and 97, respectively). We detected many more genes under positive selection in longer internal branches leading to the split of *G. atripinnis* from *A. splendens* and *X. captivus* (431 genes), *A. splendens* and *X. captivus* (404 genes), and *I. furcidens* and *X. resolanae* (579 genes). These include genes presumed to be important to the evolution of viviparity such as follicle stimulating hormone receptor (*FSHR*), androgen receptor (*AR*), vascular endothelial growth factor a (*VEGFA*), insulin growth factor 1 receptor (*IGF1R*) and insulin-degrading enzyme (*IDE*).

### Identifying genes associated with evolutionary reversal of trophotaenia development in *Ataeniobius toweri*

Based on the phylogenetic inference above and morphological evidence, the trophotaenia was likely lost in *A. toweri* following its split from *G. multiradiatus*. We, therefore, performed additional branch-site tests for positive selection on the terminal branch leading to *A. toweri*. Altogether, 1177 genes were found to be under positive selection in this branch, after correcting for multiple testing. We overlapped genes showing evidence of rapid sequence evolution in *A. toweri* with genes upregulated (log_2_ fold change > 2) in the trophotaenia of *G. multiradiatus*. We reasoned that genes undergoing rapid sequence divergence that are normally uniquely expressed in the trophotaenia may be genes associated with trophotaenia loss in *A. toweri*. We found 56 genes which showed significant upregulation in trophotaenia compared to all other tissues in *G. multiradiatus* and under positive selection in *A. toweri*. Whilst gene ontology analysis showed no significant enrichment of any terms after multiple testing correction, we note that non-significant terms relate to fin and appendage development (adjusted *p* = 0.13), consistent with a potential role for these genes in trophotaenia loss (Supplementary Table 5).

To further differentiate between trophotaenia-specific genes under positive selection in *A. toweri* for reasons other than the loss of trophotaenia, we used Variant Effect Predictor (VEP) to detect high-impact mutations that may have disruptive effects on protein structure or function. Specifically, we searched for homozygous high-impact mutations specific to *A. toweri* and overlapped these mutations with the 56 trophotaenia-specific genes (in *G. multiradiatus*) under positive selection in *A. toweri*. Only 6 genes showed evidence of high impact mutations, including a *cadherin related family member 5* (CDHR5), which modulates assembly of the intestinal brush border (Crawley et al. 2014), and *Fraser extracellular matrix complex subunit 1* (FRAS1), which is involved in epithelial-mesenchymal formation during embryonic development (Pavlakis et al. 2011).

## Discussion

Understanding the genomic dynamics of speciation is an important current challenge in evolutionary biology. Our results provide detailed genomic insight into the evolutionary divergence of *Goodeinae* and suggest a joint role for vicariant speciation with gene flow, and the importance of matrotrophic viviparity in their diversification. While the number of species we analysed is modest, they do represent all the proposed tribes of the Goodeinae and allow inference of their history. Overall, we obtained support for rapid divergence early in the evolutionary history of *Goodeinae*. We found that diversification of the *Goodeinae* began 18 MYA with the split of *Ataeniobius* and *Girardinichthys* and was followed by rapid divergence of *Characodon* and lineages including *Goodea*, *Xenotaenia*, *Xenoophorus*, *Ilyodon* and *Ameca* (representing all the tribes) around 17 MYA. These results coincide with the beginning of activity in the Trans-Mexican Volcanic Belt, and are consistent with previous phylogenetic analysis and fossil evidence (Webb et al. 2004; Ferrari et al. 2012; Foster and Piller 2018). Our results disagree with previous analysis supporting basal divergence of *Characodon* in Durango, and instead support diversification of *Goodeinae* that likely occurred east to west with divergence of *Ataeniobius* and *Girardinichthys* (Webb et al. 2004). They also do not support a grouping of *Goodea* and *Ataeniobius* in a supposed tribe Goodiini. It has been shown that the markers previously used to generate such groups vary in their phylogenetic utility and resolution (Parker et al. 2019).

Here we also demonstrate that phylogenetic conflict may also arise from incomplete lineage sorting during the early radiation, and introgression. Whilst we find complete statistical support for the early divergence events, we also find extensive underlying gene tree conflict, with (remarkably) only 12–13% of protein-coding gene trees supporting these ancestral branches. It seems likely that such gene tree conflict is generated by incomplete lineage sorting as a result of rapid early diversification. Divergence of *Xenotaenia*, *Xenoophorus*, *Ilyodon* and *Ameca* occurred 10-9 MYA, likely caused by another bout of volcanic activity beginning in the late Miocene (Ferrari et al. 2012). These divergence events coincide with an episode of volcanism beginning in the west of the Trans-Mexican Volcanic Belt around 11 MYA and migrating eastward to reach the Gulf of Mexico by 7 MYA (Ferrari et al. 2012). Whilst subsequent volcanic, and climactic, episodes through the Pliocene and Pleistocene may have facilitated within-genera diversification (Mastretta-Yanes et al. (2015)), our results imply that divergence of key lineages within *Goodeinae* likely occurred during the early geological history of the Trans-Mexican Volcanic Belt. Altogether, the phylogeographic history of *Goodeinae* inferred here suggests early divergence of the group was characterised by complex, repeated volcanic events occurring throughout the Miocene facilitating bursts of vicariant speciation, as opposed to idiosyncratic, intrinsic factors specific to each divergence event (Webb et al. 2004; Ritchie et al. 2005; Crisp and Cook 2007).

Whilst geological changes likely contributed to early diversification of *Goodeinae*, recurrent volcanic activity may have also facilitated secondary contact and gene flow. Recent work has shown that classic simple models of vicariant speciation may obscure more complex speciation histories (Kopuchian et al. 2020; Naka and Pil 2020). Here, we examined evidence of introgression between species, characterising the degree of allele sharing genome-wide and at particular genomic regions. We found limited but significant evidence of introgression between *A. toweri* and species within *Goodeini* (perhaps supporting their grouping in some analyses), *Xenotaenia*, *Xenoophorus*, *Ilyodon* and *Ameca*, which potentially represent a single gene flow event between an ancestral lineage leading to the diversification of the aforementioned species and the ancestor of *A. toweri* and *G. multiradiatus*. This is partially supported by biogeographical evidence from multiple species of *Goodeinae* likely co-occurring with *A. toweri* in the tributaries of Rio Pánuco at some point in their evolutionary history (Webb et al. 2004). Some previous analyses of genetic differentiation between populations of dimorphic and monomorphic *Goodeinae* species found gene flow to be occurring, but less readily between populations of sexually monomorphic species (Ritchie et al. 2007). Recent genetic analysis of populations within *Goodea* and *Ilyodon* suggest connectivity and gene flow between species and populations is highly variable and mediated by complex topographical changes (Beltrán-López et al. 2017, 2021). Whilst we detect moderate levels of ancient gene flow, it is difficult to tell whether gene flow is adaptive and has contributed to phenotypic diversity and diversification, as observed in other systems (Malinsky et al. 2015; Meier et al. 2017; Svardal et al. 2020).

The evolution of viviparity is thought to have driven diversification rates in *Goodeinae* (Helmstetter et al. 2016), and therefore genes involved in placental viviparity are expected to be undertaking rapid evolution (Crespi and Semeniuk 2004). At a broad level, we found limited support for this idea. Gene ontology for all genes under positive selection in at least one internal branch in *Goodeinae* show enrichment for reproduction and cilium movement. The lack of a clear link indicated by gene ontology may be due to the difficulty in detecting positive selection at short branches where incomplete lineage sorting is pervasive. We also surveyed genes under positive selection across multiple branches and found a number of canonical immune response genes including interleukin-2 receptor subunit gamma (*IL-2RG*), an essential constituent signalling component of many interleukin receptors (Russell et al. 1993); cluster of differentiation 4 (*CD4*), a central component of adaptive immune response (Luckheeram et al. 2012); and Lymphocyte activation gene 3 (*LAG3*), a gene important in modulating adaptive immune responses (Grosso et al. 2007). Positive selection on immune response genes has been shown to be a relatively common feature of surveys of positive selection across mammals, birds, and invertebrates (Jiggins and Kim 2007; Kosiol et al. 2008; Shultz and Sackton 2019). We also found evidence of introgression of genes involved in adaptive immune response between *A. toweri* and an ancestral lineage leading to the divergence of *Ameca*, *Xenoophorus*, *Goodea*, *Xenotaenia* and *Ilyodon*, which, taken with evidence of positive selection, may indicate adaptive introgression of genes involved in MHC II pathway. Recurrent positive selection on immune-related genes, particularly genes involved in MHC II pathway, have also been associated with the evolution of viviparity in pipefish and seahorses (Roth et al. 2020), and broadly across Cyprinodontiformes (Yusuf et al. 2023).

In specific branches within the *Goodeinae*, we identified genes known to be important in viviparity under positive selection. For example, we found positive selection in *ar* and *fshr*, well known mediators of hormone synthesis and regulation in mammalian viviparity and reproduction. We also found evidence of positive selection on *igfr1*, but not *igfr2*, where parent-of-origin expression has been shown to mediate drastic changes in offspring size and positive selection has been observed in placental fish (DeChiara et al. 1990, 1991; O’Neill et al. 2007). Saldivar Lemus et al. (2017) showed that similar parent-of-origin methylation effects on *igf2* are also present in *G. multiradiatus*, suggesting genes mediating sexual conflict over offspring size may be under rapid evolution within *Goodeinae*.

Additionally, our phylogenetic analysis suggests an evolutionary reversal in the form of trophotaenia loss in *A. toweri* (Meek 1904). This is also supported by other phylogenetic analysis of mitochondrial sequences that place *A. toweri* within Goodeinae, and specifically not at the base of the Goodeinae (Webb et al. 2004; Doadrio and Domínguez 2004; Foster and Piller 2018). There have been few attempts to explain the position of *A. toweri* in Goodeinae or the hypothesised loss of trophotaenia. One potential explanation for the trophotaenia loss is the resolution of ongoing genomic conflict between maternal resource allocation to offspring, versus exploitation of resources by offspring through the expression of the paternal genome (Trivers 1974; Crespi and Semeniuk 2004; Saldívar-Lemus and Macías Garcia 2022). In Goodeids, the trophotaenial placenta contains a maternal and embryonic component and allows maternal provisioning of offspring, resulting in increases up to 37,800% in embryonic dry weight (Lombardi and Wourms 1979; Schindler 2005; Uribe et al. 2005). In mammals, discrepancies between maternal and foetal optima are thought to have led to manipulation of maternal provisioning by offspring (Haig 1993, 1996).

Whilst mother-offspring conflict has not yet been directly studied in Goodeids, cannibalism on developing siblings has evolved in more than one species (Greven and Grossherr 1992; Saldivar Lemus pers. obs.). Additionally, a potential genomic mechanism for conflict has been identified in *G. multiradiatus* (Saldivar Lemus et al. 2017), suggesting that mother-offspring conflict may be at play, alongside strong hypothesised sexual conflict (Ritchie et al. 2005, 2007). Under the phylogenetic scenario proposed in this study, loss of trophotaenia in *A. toweri* may result in mothers regaining control over resource allocation. In trying to understand the genes that may have been involved in the loss trophotaenia in *A. toweri*, we inferred positive selection in the terminal branch leading to *A. toweri* and also identified genes that were upregulated in the trophotaenia of *G. multiradiatus* (Du et al. 2022). Whilst we found no significant enrichment of any gene ontology terms, we did find some genes involved in fin and appendage development, suggesting a potential link between genes identified using our approach and morphological change in *A. toweri*. However, genes implicated in trophotaenia may also be under relaxed selection following loss of this structure leading to elevated gene-wide divergence (Hiller et al. 2012; Wertheim et al. 2015).

## Conclusion

Geological and climactic changes can have profound effects on demography, often resulting in complex speciation histories. The *Goodeinae* radiated rapidly during the Miocene likely driven by periodic volcanism. Volcanic activity and changes in species distribution facilitated ancient gene flow between ancestral lineages. We show that the same introgressed genetic variation is maintained in *A. toweri, and G. atripinnis, X. captivus* and *A. splendens*, perhaps hinting at a potential adaptive role for gene flow during the radiation of the group. Genes repeatedly under positive selection likely play a role in the evolution of matrotrophic viviparity, and we highlight specific genes that may be implicated in the loss or poorly developed trophotaenia in *A. toweri*. This study highlights the importance of environmental factors, alongside internal factors such as viviparity, in driving species divergence, and lays the groundwork for understanding the evolution of viviparity in light of the complex demographic changes that have occurred in *Goodeinae*.

### Supplementary information

Supplementary Table 1

Supplementary Table 2

Supplementary Table 3

Supplementary Table 4

Supplementary Table 5

Supplementary Table 6

Supplementary Table 7

Supplementary Methods and Materials.

## Data Availability

Scripts used to generate the assemblies and annotation can be found here: https://github.com/peterthorpe5/fish_genome_assembly. Scripts used in the comparative analysis of convergent evolution can be found here: https://github.com/LeebanY/GoodeidsPhylogenomicsComplexDemography. Genomes and whole-genome sequencing reads are available at the NCBI under the following BioProject accessions: PRJNA732843, PRJNA745519, PRJNA734141, PRJNA733895, PRJNA733912, PRJNA734011, PRJNA734170, PRJNA734774, PRJNA734771.
