# Supplementary material for: Evidence for gene flow and trait reversal during radiation of Mexican Goodeid fish
Source: Heredity (Edinb). 2024 Jun 10;133(2):78–87. doi: 10.1038/s41437-024-00694-1 (PMC11286751; doi:10.1038/s41437-024-00694-1)
Supplement: Supplementary file 8 — Supplementary Methods and Materials. [file 41437_2024_694_MOESM8_ESM.docx]

# Supplementary Material

**Methods**

**Sampling details**

After collection, fish were selected and put into plastic bags (3 fish per bag*; G. atripinnis, X. resolanae, X. captivus, A. splendens and I. furcidens*) or into plastic containers (3 fish per container; *A. splendens and I. furcidens*) with local water, Stress Coat ®, potassium permanganate and zeolite. Bags or plastic containers were then put within coolers with local water and ice to decrease fish’s metabolism during transportation to the Aquarium of the Institute of Ecology, UNAM. When transportation to the aquarium took more than few hours, faeces were removed using a pipette and more water with Stress Coat ®, potassium permanganate and zeolite was added. Once in the aquarium, fish were acclimatised by leaving the unopened bags or plastic containers for 15 minutes into quarantine tanks until the temperature of the water in the bags/containers and tank was similar. Tanks were previously prepared with stress coat, potassium permanganate, Aquari-Sol ®, and aquarium salt. Once fish were climatised, they were gently released into the tanks and fed the next day. Fish were checked twice daily for signs of infections and treated accordingly.

**Genome assemblies**

Genome assemblies for I. furcidens, *A. splendens, A. toweri* and *C. lateralis* were performed. Raw reads from whole-genome sequencing were assessed for quality using FastQC (Andrews, 2010). Reads were subsequently quality trimmed using Trimmomatic (version 0.38). Trimmed reads were then assembled using spades (version 3.14.1) (Prjibelski et al., 2020). We used Blobtools version 1.0 (Laetsch and Blaxter, 2017) to identify and remove contaminant contigs. Contigs identified as fungal, bacterial, plantal or viral were removed, yielding a contamination free final unpolished assembly. The assemblies were then scaffolded using one iteration of SSPACE (v3.0) (Boetzer et al., 2011) with BWA for alignment. Finally, we performed three iterations of Pilon (version 1.23) (Walker et al., 2014) to polish the assemblies. At all stages of assembly BUSCO (Simão et al., 2015) version 1.1b was used to assess the relative completeness of the assembled genomes using Eukaryotic odb9 models. Separately, whole-genome sequencing data for *X. captivus, G. atripinis, X. resolanae* and *C. baileyi* generated in (Yusuf et al. 2023) were also used in this study. Finally, whole-genome sequencing data and the highly-contiguous genome assembly of Girardinichthys multiradiatus from (Du et al., 2022) were used as reference for read mapping and phylogenetic analyses.

**Read filtering**

Variant calling was performed on mapped reads using Freebayes (v. 1.3.2) using the following parameters: --report-genotype-likelihood-max --no-population-priors --use-best-n-alleles 4 --hwe-priors-off --use-mapping-quality --ploidy 2 --theta 0.02 --haplotype-length -1 --genotype-qualities. Variants were filtered using GATK hard-filtering best practices guidelines (Van der Auwera et al., 2013) (QD < 2.0, QUAL < 30.0, SOR > 3.0, FS > 60.0, MQ < 40.0, MQRankSum < -12.5, ReadPosRankSum < -8.0).

**Dating of Goodeidae divergence times.**

To generate our selectively-neutral non-coding dataset, we first estimated substitution rates of four-fold degenerate sites (non-conserved regions) in 21 species across *Cyprinodontiformes* to identify regions with conserved substitution rates using PhastCons (Siepel *et al.*, 2005; Pollard *et al.*, 2010; Yusuf *et al.*, 2023). These species include: *Xenotaenia resolanae, Xenoophorus captivus, Goodea atripinnis, Crenicthys baileyi, Poecilia reticulata, Cyprinodon nevadensis, Gambusia affinis, Gambusia holbrooki, Poeciliopsis turrubarensis, Poeciliopsis retropinna, Poeciliopsis occidentalis, Orestias ascotanensis, Cyprinodon variegatus, Xiphophorus maculatus, Poecilia mexicana, Poecilia latipinna, Poecilia formosa, Xiphophorus hellerii, Xiphophorus couchianus, Fundulus heteroclitus, Girardinicthys multiradiatus.* Variants falling within these conserved regions were subsequently removed from our dataset, alongside coding regions using bedtools (v. 1.12) (Quinlan and Hall, 2010). Additionally, to ensure sampled loci represented different evolutionary histories as required by BPP, we made 10kb windows that were at least 100kb away from each other. This yielded 2,740 loci of variable size that were converted into alignments with no missing sites for any species using vcf2phylip (Ortiz, 2019). To estimate divergence times and effective population sizes, we used A00 analysis with default settings except for amendments to theta (0.003) and tau (0.03) priors. We used burn-in periods of 8,000 and 100,000 samples. Divergence time estimates and effective population size estimates were scaled to geological time using the *Xiphophorus* *maculatus* mutation rate (3.5 x 10^-9^) from (Schumer *et al.*, 2018) and a generation time of one via R package bppr (v.0.6.1) (Angelis and Dos Reis, 2015).

**F-branch statistic.**

Because introgression signals can represent both very recent introgression between extant lineages and ancient introgression between ancestral lineages, we inferred the *f-branch* statistic via Dsuite. The *f-branch* statistic computes allele sharing using *f4-ratios* between P3, where P3 is an outgroup species to P1 species and P2 species, and the descendants of a branch labelled *b* (i.e., the descendants of P2, for example) relative to a sister branch labelled *a*. This statistic allows for correlated *f4-ratios* to be disentangled and specific branches with strong introgression signals to be identified.


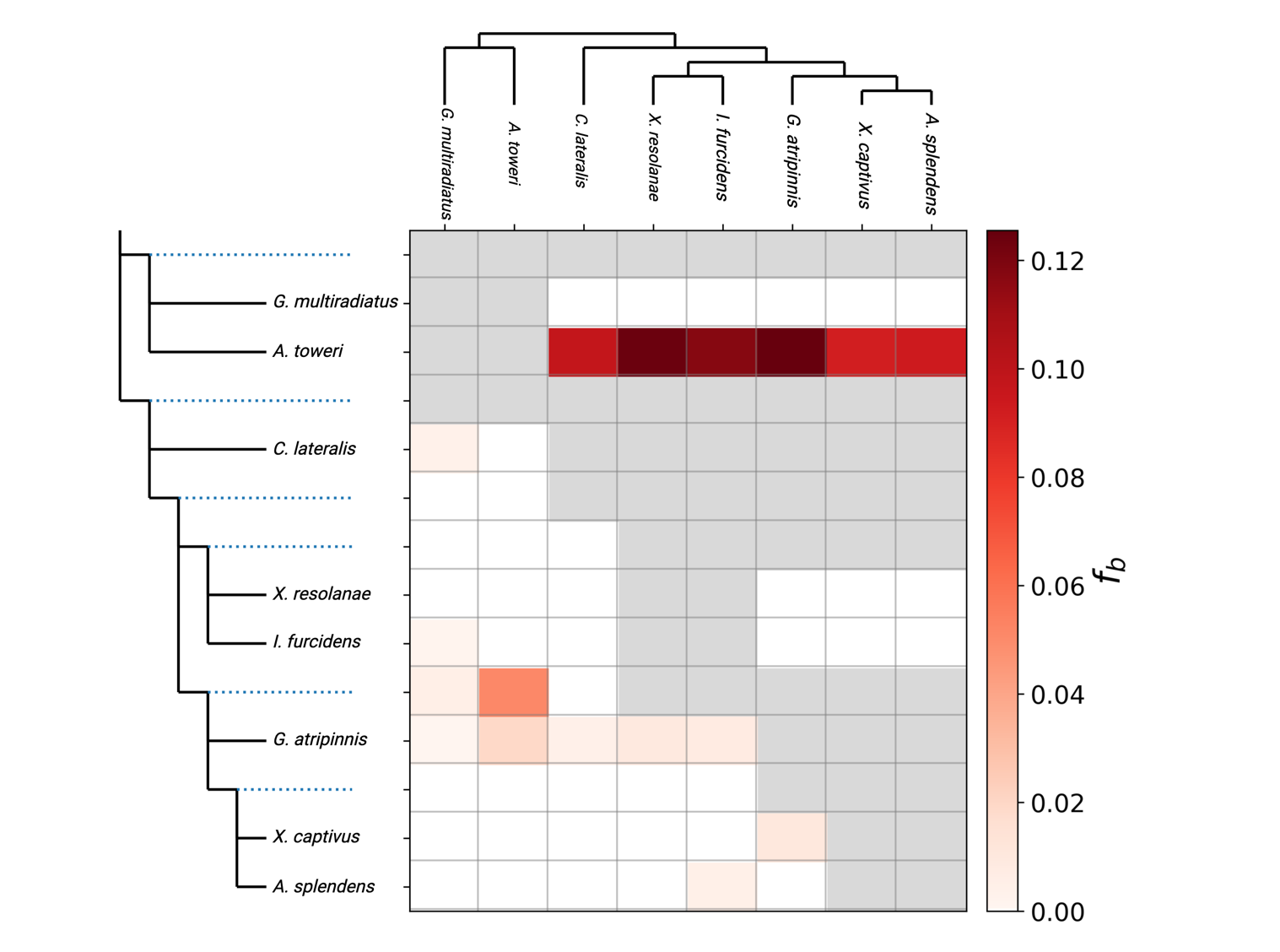
**Supplementary Figures**

**Supplementary Figure 1: F-branch statistics across Goodeinae.** *Phylogeny on the top represents the species tree phylogeny, and the phylogeny on the side represents the species tree phylogeny with ancestral nodes denoted by the dashed lines. Grey squares are squares where f_b_*  statistic values were not significant.

**Supplementary Figure 1: F-branch statistics across Goodeinae.** Phylogeny on the top represents the species tree phylogeny, and the phylogeny on the side represents the species tree phylogeny with ancestral nodes denoted by the dashed lines. Grey squares are squares where f_b_ statistic values were not significant.

**Supplementary Figure 2: Sampling locations for species included in this study.** X. resolanae and I. furcidens were sampled in the same site, and similarly, A. toweri and G. multiradiatus were also sampled at the same site.

## Supplementary References

1. Angelis K, Dos Reis M (2015). The impact of ancestral population size and incomplete lineage sorting on Bayesian estimation of species divergence times. *Current Zoology* **61**: 874–885.
2. Ortiz EM 2019 (2019). *vcf2phylip v2.0: convert a VCF matrix into several matrix formats for phylogenetic analysis. (Zenodo).*
3. Pollard KS, Hubisz MJ, Rosenbloom KR, Siepel A (2010). Detection of nonneutral substitution rates on mammalian phylogenies. *Genome Research*.
4. Quinlan AR, Hall IM (2010). BEDTools: A flexible suite of utilities for comparing genomic features. *Bioinformatics*.
5. Schumer M, Xu C, Powell DL, Durvasula A, Skov L, Holland C, *et al.* (2018). Natural selection interacts with recombination to shape the evolution of hybrid genomes. *Science*.
6. Siepel A, Bejerano G, Pedersen JS, Hinrichs AS, Hou M, Rosenbloom K, *et al.* (2005). Evolutionarily conserved elements in vertebrate, insect, worm, and yeast genomes. *Genome Research*.
7. Yusuf LH, Saldívar Lemus Y, Thorpe P, Macías Garcia C, Ritchie MG (2023). Genomic Signatures Associated with Transitions to Viviparity in Cyprinodontiformes. *Molecular Biology and Evolution* **40**: msad208.
8. Angelis K, Dos Reis M (2015). The impact of ancestral population size and incomplete lineage sorting on Bayesian estimation of species divergence times. *Current Zoology* **61**: 874–885.
9. Ortiz EM 2019 (2019). *vcf2phylip v2.0: convert a VCF matrix into several matrix formats for phylogenetic analysis. (Zenodo).*
10. Pollard KS, Hubisz MJ, Rosenbloom KR, Siepel A (2010). Detection of nonneutral substitution rates on mammalian phylogenies. *Genome Research*.
11. Quinlan AR, Hall IM (2010). BEDTools: A flexible suite of utilities for comparing genomic features. *Bioinformatics*.
12. Schumer M, Xu C, Powell DL, Durvasula A, Skov L, Holland C, *et al.* (2018). Natural selection interacts with recombination to shape the evolution of hybrid genomes. *Science*.
13. Siepel A, Bejerano G, Pedersen JS, Hinrichs AS, Hou M, Rosenbloom K, *et al.* (2005). Evolutionarily conserved elements in vertebrate, insect, worm, and yeast genomes. *Genome Research*.
14. Yusuf LH, Saldívar Lemus Y, Thorpe P, Macías Garcia C, Ritchie MG (2023). Genomic Signatures Associated with Transitions to Viviparity in Cyprinodontiformes. *Molecular Biology and Evolution* **40**: msad208.
